# A new kind of auxiliary heart in insects: functional morphology and neuronal control of the accessory pulsatile organs of the cricket ovipositor

**DOI:** 10.1186/1742-9994-11-43

**Published:** 2014-06-08

**Authors:** Reinhold Hustert, Matthias Frisch, Alexander Böhm, Günther Pass

**Affiliations:** 1 Department of Neurobiology, JFB-Institute for Zoology, University of Göttingen, Berliner Straße 28, 37073 Göttingen, Germany; 2 Department of Integrative Zoology, University of Vienna, Althanstraße 14, 1090 Vienna, Austria

**Keywords:** Orthoptera, Gryllidae, Abdomen, Circulation, Hemolymph, Neuroanatomy, Neurogenic, Terminal ganglion, Central pattern generator, Evolutionary novelty

## Abstract

**Introduction:**

In insects, the pumping of the dorsal heart causes circulation of hemolymph throughout the central body cavity, but not within the interior of long body appendages. Hemolymph exchange in these dead-end structures is accomplished by special flow-guiding structures and/or autonomous pulsatile organs (“auxiliary hearts”). In this paper accessory pulsatile organs for an insect ovipositor are described for the first time. We studied these organs in females of the cricket *Acheta domesticus* by analyzing their functional morphology, neuroanatomy and physiological control.

**Results:**

The lumen of the four long ovipositor valves is subdivided by longitudinal septa of connective tissue into efferent and afferent hemolymph sinuses which are confluent distally. The countercurrent flow in these sinuses is effected by pulsatile organs which are located at the bases of the ovipositor valves. Each of the four organs consists of a pumping chamber which is compressed by rhythmically contracting muscles. The morphology of the paired organs is laterally mirrored, and there are differences in some details between the dorsal and ventral organs. The compression of the pumping chambers of each valve pair occurs with a left-right alternating rhythm with a frequency of 0.2 to 0.5 Hz and is synchronized between the dorsal and ventral organs. The more anteriorly located genital chamber shows rhythmical lateral movements simultaneous to those of the ovipositor pulsatile organs and probably supports the hemolymph exchange in the abdominal apex region. The left-right alternating rhythm is produced by a central pattern generator located in the terminal ganglion. It requires no sensory feedback for its output since it persists in the completely isolated ganglion. Rhythm-modulating and rhythm-resetting interneurons are identified in the terminal ganglion.

**Conclusion:**

The circulatory organs of the cricket ovipositor have a unique functional morphology. The pumping apparatus at the base of each ovipositor valve operates like a bellow. It forces hemolymph via sinuses delimited by thin septa of connective tissue in a countercurrent flow through the valve lumen. The pumping activity is based on neurogenic control by a central pattern generator in the terminal ganglion.

## Introduction

In the open circulatory system of insects, the pumping dorsal heart tube circulates hemolymph in the central body cavity enabling a constant perfusion of the internal organs and tissues. This flow, however, cannot effect circulation in outlying dead-end structures, such as antennae, legs, wings and abdominal appendages. For this task, insects have special hemolymph guiding structures and/or auxiliary hearts [1-3].

In appendages, such as the thoracic legs and some abdominal appendages, a longitudinal septum divides the lumen into two sinuses. Distally the septum is lacking, and the sinuses are confluent. Thereby a countercurrent flow is enabled within these appendages, and we distinguish between an efferent and afferent sinus. How the hemolymph flow is produced remains unclear in most cases [3,4]. In some appendages, pressure changes due to regular volume alterations of tracheae or tracheal sacs contribute to the hemolymph exchange [5,6]. More elaborate organs for the supply of hemolymph to long body appendages are the so-called accessory pulsatile organs or auxiliary hearts. These muscle-driven pumps can be very diverse in their functional morphology in the various groups of insects. They may be located at the base or within the appendages and are in general autonomous organs which pump rhythmically, but independently, from that of the dorsal heart. The contractions of these auxiliary hearts are based on a myogenic automatism which can be modulated by neuronal and/or neurohormonal control [7-10]. A thoroughly investigated example of such an auxiliary heart is the antenna-heart of the cockroach *Periplaneta americana* in which the functional morphology, neuroanatomy, neurochemistry, pharmacology and the control mechanisms have been analyzed in detail [11-16].

However, the problem of circulation has not yet been investigated in insect ovipositors although some of them reach considerable length. In this paper we describe for the first time accessory pulsatile organs for these body appendages. The organs were discovered in the female cricket *Acheta domesticus* (preliminary notes [1,17]). In live specimens, hemocyte movements can be observed under the microscope through transparent parts of the ovipositor cuticle. The flow occurs in pulses that are clearly correlated with conspicuous compressions of structures at the base of the ovipositor valves which were revealed to be the pumping organs for hemolymph circulation in these appendages. The functional morphology of these ovipositor pulsatile organs was investigated on the basis of serial semi-thin sections and a microCT scan in combination with *in vivo* observations. In addition, neuroanatomical and physiological studies were performed. Several motoneurons and interneurons involved in the control of the ovipositor pulsatile organs could be identified in the terminal ganglion. The electrophysiological recordings revealed a coordinated and rhythmic bilateral motor output from these neurons. Since the rhythm persists even when the terminal ganglion is completely isolated, it could serve as a model for studies of autonomous rhythm generation in a neural network (preliminary reports [17,18]).

## Results

### Ovipositor and anatomical condition at the abdominal apex

**Figure 1:**
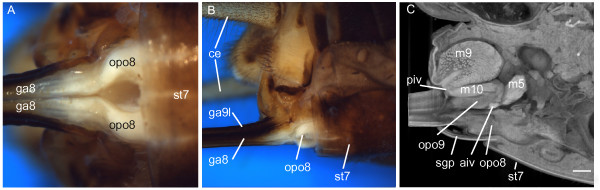
**Location of the ovipositor pulsatile organs in the abdominal apex. (A)** Ventral view (subgenital plate removed): the distally strongly sclerotized paired gonapophyses 8 (ga8) have proximally an extensive area of thin and flexible cuticle. The distinct bulges at their basal articulation represent the ovipositor pulsatile organs 8 (opo8). **(B)** Lateral view (subgenital plate removed): the ovipositor shaft consists of the ga8 and the lateral gonapophysis 9 (ga9l); opo8 form protruding bulges. **(C)** Volume rendering of microCT data in lateral view showing spatial relationship of opo8 and opo9 to the anterior and posterior intervalvular sclerites (aiv, piv) and some larger muscles (numbering follows [19]). Scale bar: 500 μm. Further abbreviations: ce, cercus; sgp, subgenital plate; st7, sternite of the 7^th^ abdominal segment.

The ovipositor shaft is composed of four long valves: a ventral pair, referred to as the gonapophyses of the abdominal segment 8 (ga8; synonyms: 1^st^ valvulae of Snodgrass [19,20], 1^st^ gonapophyses of Scudder [21]), and a dorsal pair, referred to as the lateral gonapophyses of the abdominal segment 9 (ga9l; 3^rd^ valvulae of Snodgrass [19,20], gonoplacs of Scudder [21]). The median gonapophyses of the 9^th^ segment (ga9m, synonyms: 2^nd^ valvulae of Snodgrass [19,20], 2^nd^ gonapophyses of Scudder [21]) are very short and inconspicuous structures in crickets. The long ovipositor valves are distinctly widened at their bases and interconnected by several joints to the highly modified and strongly sclerotized coxosternites of the abdominal segment 8 and 9, respectively (cs8 and cs9; synonyms: 1^st^ and 2^nd^ valvifers of Snodgrass [19,20], gonocoxae of Scudder [21], coxosternite of Klass and Ulbricht [22]). The lumen of the four long ovipositor valves is subdivided by delicate septa of connective tissue into sinuses in which efferent or afferent flows of hemolymph can be observed in live specimens. Aside from the epidermis, the ovipositor valves only contain nerves and few tracheal trunks extending to the apical tip. The ovipositor pulsatile organs (opo) are located at the base of the appendages (Figure 1).

### Ovipositor pulsatile organs of the abdominal segment 8

**Figure 2:**
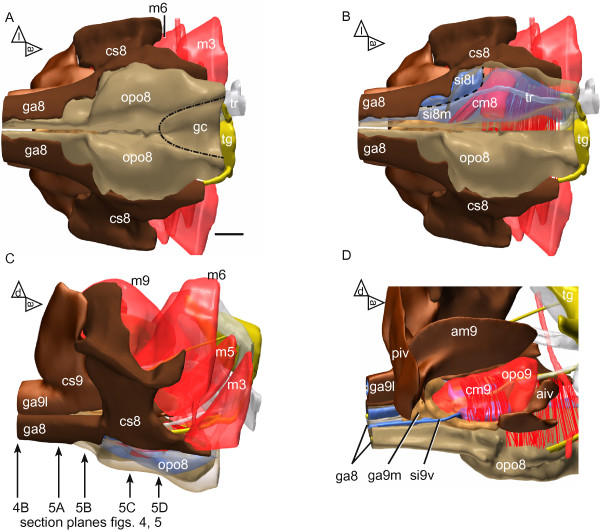
**Reconstruction of the ovipositor pulsatile organs and the surrounding structures of the abdominal apex. (A)** Ventral view (subgenital plate). Bases of the gonapophyses 8 (ga8) enlarged and with strongly sclerotized lateral parts (*dark brown*), medial parts of flexible cuticle (*light brown*) forming compressible bulges which constitute the ovipositor pulsatile organ 8 (opo8); area of the preceding genital chamber (gc) delimited by dotted line. **(B)** Same view as in A, but right ga8 base is presented transparently; compressor muscle 8 (cm8) (*red*) extends between strongly sclerotized coxosternite 8 (cs8) and medial flexible cuticle of ga8; lumen of ga8 is divided by a delicate septum (indicated by dashed line) into a wide medial sinus (si8m) and a smaller lateral sinus (si8l; both sinuses in *blue*) **(C)** Lateral view. ga8 and lateral gonapophysis 9 (ga9l) are articulately jointed to cs8 and cs9. The bulges of the protruding ovipositor pulsatile organ (opo8) are presented transparently. Arrows refer to section levels of Figures 4 and 5. **(D)** Parasagittal view of the opo, most of left cs9 and many large muscles are removed to expose opo9; cm9 extends between cs9 and a median invagination of flexible cuticle. Scale bar: 250 μm. Further abbreviations (numbers 8 and 9 refer to the respective abdominal segment): aiv, anterior intervalvular sclerite; am, median apodeme (attachment site of muscle 9); m, muscle (numbering follows [19]); piv, posterior intervalvular sclerite; tr, trachea; tg, terminal ganglion. Coloring of structures in italics. Arrowheads outside the diagram indicate reference planes: a, anterior; d, dorsal; l, lateral.

**Figure 3:**
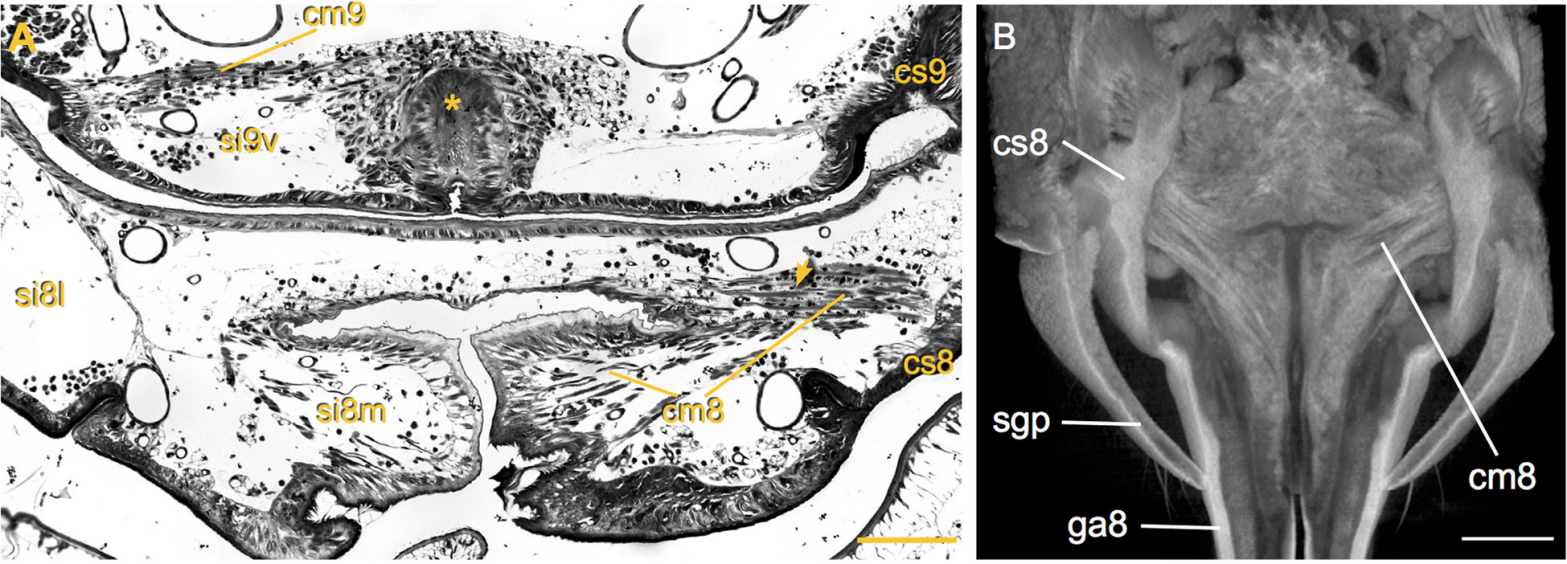
**Attachments and course of pulsatile organ compressor muscles. (A)** Cross section through ventral part of abdomen (freshly molted female mostly free of fat tissue, muscle strands therefore clearly visible). Ovipositor pulsatile organ 8: compressor muscle 8 (cm8) extends between coxosternite 8 (cs8) and median flexible cuticle; yellow arrow indicates muscle strands which are attached to the flat cuticular area between the compressible bulges. Ovipositor pulsatile organ 9: compressor muscle 9 (cm9) extends between coxosternite 9 (cs9) and median cuticular invagination which is flexible at the basis and at the innermost part forms a strongly sclerotized structure (asterisk). **(B)** Volume rendering from microCT data with ventral view of gonapophyses 8 (ga8) bases. The cm8 muscle strands originate laterally at the cs8, fan out extending through the medial sinus (si8m) and are attached at the medial flexible cuticle of ga8 base. Scale bar: **(A)** 100 μm, **(B)** 450 μm.

**Figure 4:**
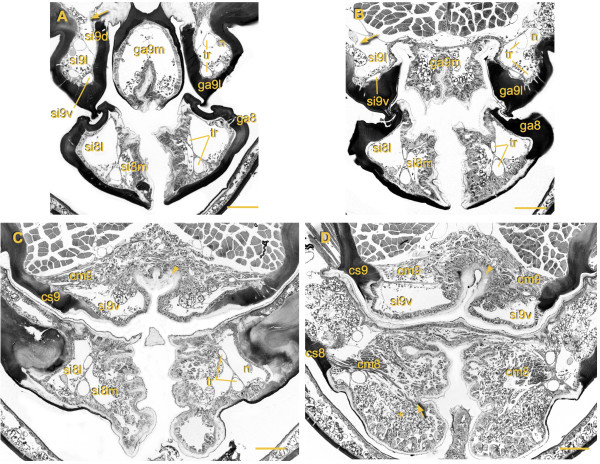
**Organization of hemolymph sinuses in the ovipositor and their fusion with the abdominal hemocoel.** Cross sections from distal to proximal, section levels indicated in figure 2C by arrows. **(A)** Gonapophysis 8 (ga8): note strong sclerotized lateral and flexible medial cuticle, lumen divided by septum with associated tracheae (tr) into efferent medial sinus (si8m) and afferent lateral sinus (si8l); lateral gonapophysis 9 (ga9l): efferent ventral sinus (si9v), afferent lateral sinus (si9l) and afferent dorsal sinus (si9d); the latter merges on the left side with the abdominal hemocoel (arrow); medial gonapophysis 9 (ga9m): each valve with undivided lumen; **(B)** left afferent si9l merges with abdominal hemocoel. **(C)** Ovipositor pulsatile organ 8: si8m partly filled with fat tissue; ovipositor pulsatile organ 9: the two pumping chambers are separated by invagination of ventral cuticle, compressor muscle 9 (cm9) originates laterally at coxosternite 9 (cs9) and extends to the medial cuticular invagination (arrowhead). **(D)** Ovipositor pulsatile organ 8: cm8 originates laterally at cs8, fans out into individual strands which extend to attachment site at medial flexible cuticle (arrow), in-between fat tissue (asterisk); ovipositor pulsatile organ 9: cm9 dorsal of si9v extending between cs9 and strong sclerotized part of medial cuticular invagination (arrowhead). Scale bars: 100 μm.

Removal of the subgenital plate exposes the bases of the ga8 (Figure 1A, B; Figure 2A). Their lateral sides consist of a strongly sclerotized cuticle while medially the cuticle is thin and flexible forming a conspicuous bulge at each ga8 base (“soft lateral walls” of [23]). The two bulges form the soft-walled margin of the genital opening. In live specimens conspicuous movements of the bulges can be observed: they are strongly compressed and expanded in an alternating left-right rhythm of 0.2-0.5 Hz at room temperature (see Additional file 1 video). Our study concludes that the compressible bulges constitute the pumping chambers of the ovipositor pulsatile organs of abdominal segment 8 (opo8). They serve as bellow-like pumping devices for hemolymph transport through the ga8. The compression is caused by a muscle that extends across the lumen of the bulges (cm8, Figure 2B). The muscle originates laterally at the sclerotized cs8 and fans out into individual strands which attach medially to the soft wall of the bulge (Figures 3A, B; Figure 4D). Contraction of this muscle pulls the soft parts of the cuticle in a lateral direction. Thereby the pumping chamber is compressed, and hemolymph is forced into the ga8. The individual muscle strands are extended at slightly different angles enabling compression of a wide area of the bulge during contraction. The compression of one bulge supports the dilation of the other bulge and thereby helps to fill it with hemolymph drawn from the abdominal body cavity. In this way the two opo8 operate as two partly interconnected left-right alternating bellows.

After each pumping stroke the hemolymph slightly flows back before it stalls. This observation is considered an indication for the absence of a discrete valve device within the ga8. However, on each body side some of the more anterior and dorsal muscle strands of the cm8 are attached to the lateral margins of the flat cuticle part between the two bulges (Figure 3A, arrow). During bulge compression this part is pulled strongly into a lateral direction thereby narrowing the base of the bulge. This movement may partly prevent backflow of hemolymph into the abdominal cavity.

**Figure 5:**
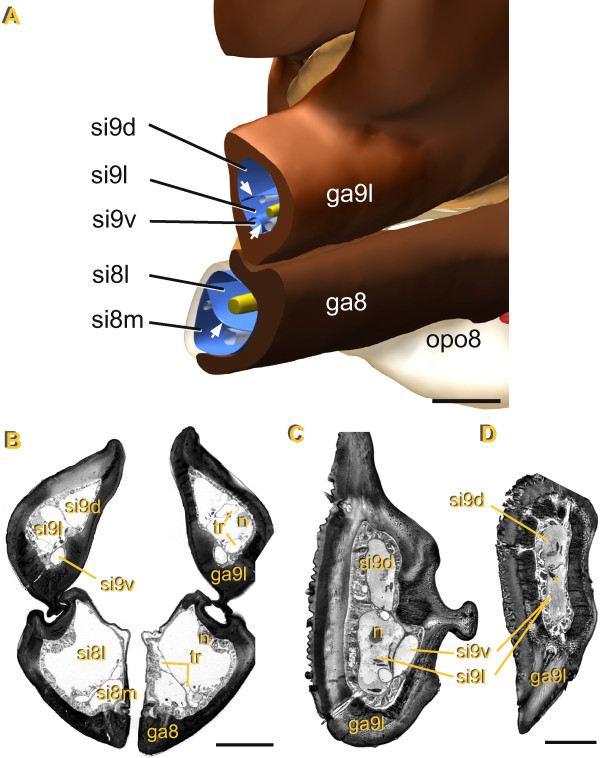
**Morphology and inner organization of the gonapophyses. (A)** Reconstruction of right half of ovipositor. Aside from epidermis, gonapophyses contain nerves (yellow), tracheae (gray) and hemolymph; their lumen is divided by thin septa of connective tissue (arrows) into hemolymph sinuses guiding the countercurrent flow; gonapophysis 8 (ga8) with efferent medial sinus (si8m) and afferent lateral sinus (si8l), gonapophysis 9 (ga9) with efferent lateral sinus (si9l), afferent dorsal sinus (si9d) and afferent ventral sinus (si9v) **(B)** Cross section through paired ga8 and ga9, same level as in diagram above (n: nerves, tr: tracheae). **(C, D)** Cross sections through most distal region of right ga9, si9v and si9l in **(C)** merge into one sinus **(D)**, asterisk indicates hemocyte moving through septum gap into neighboring sinus. Scale bar: **(A)** 150 μm, **(B)** 100 μm, **(C, D)** 50 μm.

The lumen of each bulge is divided by a septum into a wide median sinus (si8m) which contains the cm8 in its proximal portion, and a narrower lateral sinus (si8l) which is devoid of any muscles (Figure 3A; Figure 4C). The septum extends into the ga8 nearly up to the apex and separates the somewhat smaller si8m from the larger si8l (Figure 5A, B). Compressions of the opo8 force hemolymph into a distal direction within the si8m. Observed peak flow velocities of hemocytes range between 5 to 15 mm.s^-1^, and hemolymph can thereby be moved from the base to the apex of the ga8 with a single pumping stroke. At the lancet-like apex of the ga8, the septum is perforated and the hemolymph can flow into the lateral sinus. There it returns to the base of the ga8 and continues further back dorso-laterally into the body cavity.Two tracheae are embedded within the septum which separates the si8m and si8l in each ga8 (Figure 4C, D). During each pumping stroke these tracheae become laterally displaced and partly compressed. When the ipsilateral bulge relaxes, the tracheae return to their original shape indicating that during pumping strokes the hemolymph pressure in the si8m is higher than in the si8l.

### Ovipositor pulsatile organs of the abdominal segment 9

The pump system of the ovipositor pulsatile organs 9 (opo9) overlies the posterior part of the opo8 (Figure 2C, D). Its functional principle resembles that of the abdominal segment 8, but there are some anatomical differences which may be explained by the deeper integration of the ga9 bases into the abdominal apex. The soft cuticular parts of the opo9 (Figure 4C, D) extend ventrally between the lateral strongly sclerotized parts of the coxosternite 9 (cs9), as well as the anterior and posterior intervalvular sclerites (aiv and piv after Snodgrass [19], Figure 2D). Medially, the walls of the two ga9 bases are narrowly apposed which appears from outside as slit-like invagination (Figure 4A, B). It consists for the most part of flexible cuticle but in the midline, where the right and left ga9 meet, there is a strongly sclerotized structure (Figure 3A). The small hemocoel spaces lateral of the invagination represent the pump chambers of opo9. Each chamber is continuous with the ventral sinus (si9v) of the ipsilateral ga9 (Figures 2A, 3A, 4 and 5), and dorsally each chamber is covered by a muscle (cm9) that is attached to the upper part of the invagination and laterally to the cs9 (Figure 3A; Figure 4C, D). In live specimens one can observe that alternating contractions of the left and right muscles tilt the median cuticular structure and the flexible median cuticle portion to the corresponding side (see Additional file 1: Video). Thereby the two pump chambers are compressed and widened in alternation and hemolymph is forced into the si9v of each ga9l. The opo9 operates, similar to the opo8, as a pair of interconnected left-right alternating bellows: compression of one pump chamber (systole) simultaneously widens the opposite chamber (diastole) and stretches its compressor muscles (cm9). This leads to contraction of the cm9 thereby completing a full pumping cycle.Hemolymph flows through the si9v of ga9l to the apex and passes through small gaps in the septa to si9d and si9i (Figure 5C, D). From these two sinuses the hemolymph flows back to the ovipositor base. During each pumping stroke the tracheae in the si9d of the ga9l (Figure 4B) become displaced and partly collapse; in the intervals they return to their original position.

### Genital chamber movements

**Figure 6:**
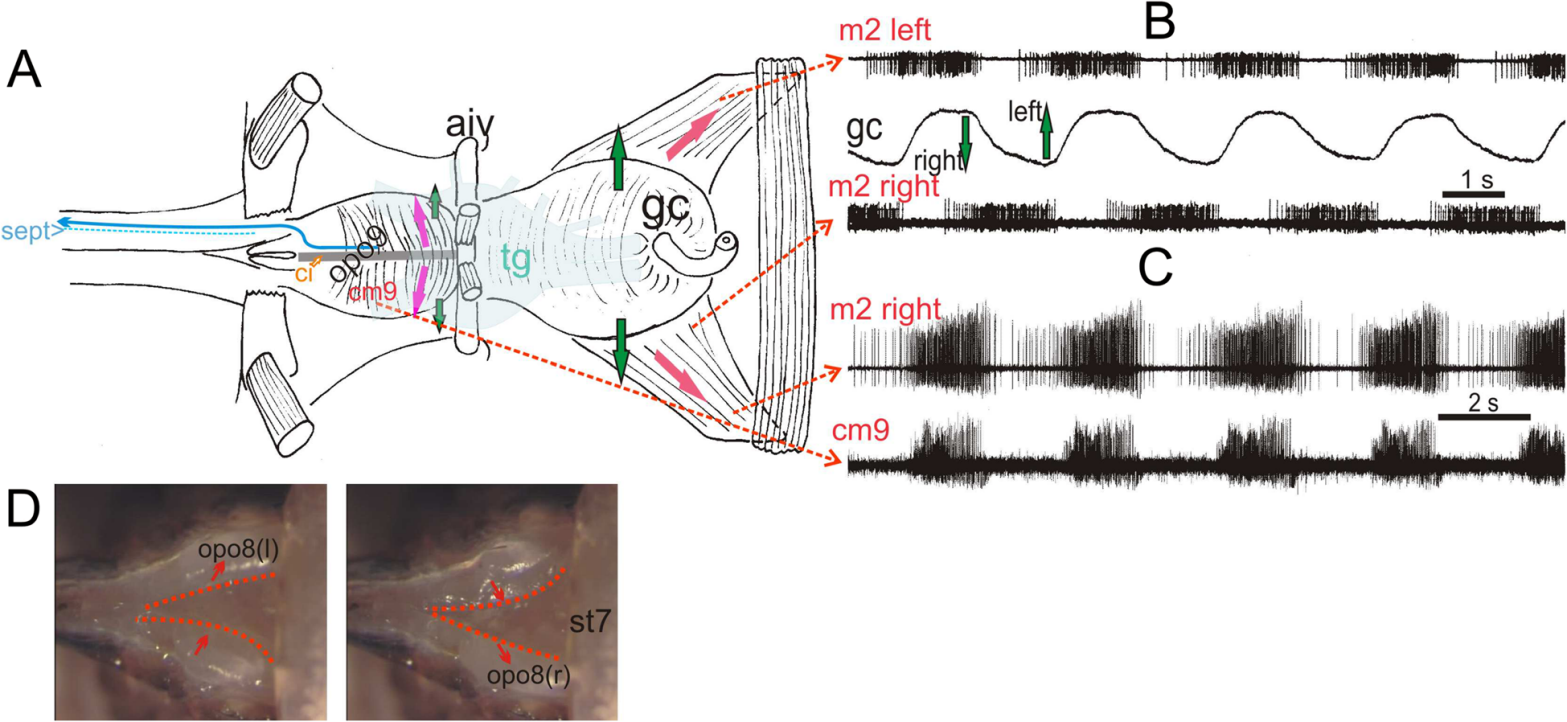
**Genital chamber and ovipositor pulsatile organs**, **their movements and pumping activity. (A)** Schematic dorsal view of genital chamber (gc) and ovipositor pulsatile organ 9 (opo9) diagramming their synchronous tilting (green arrows) due to contractions of the muscles m2 and compressor muscle (cm9, red arrows). This results in lateral tilting of the internal cuticular ridge (ci, grey bar) and hemolymph flow into the ipsi-lateral sinus 9 (si9l, blue arrow); overlying tg outlined in light blue. **(B)** Bursting pattern of the anterior muscle pair (m2) of the gc during left-right tilting (middle trace, arrows) recorded from their surface by suction electrodes; movements of gc recorded with a piezoelectric tongue. **(C)** Synchronous rhythm of gc and opo9 recorded ipsilaterally from the muscle surfaces (stippled arrows). **(D)** Ventral view of the ovipositor pulsatile organ 8 (opo8) after removal of the subgenital plate up to sternite 7 (st7) with the two alternating stages of left-right contraction (outlined with red stippling). The median part of the soft-walled bulge is drawn laterally (hemolymph pressurized inside) while that of the other bulge expands at the same time medially (hemolymph drawn in from abdominal hemocoel).

The regular pumping of the left and right opos is well synchronized with a rhythmic bilateral movement of the genital chamber (gc) located in the posterior ventral abdomen (Figure 6). It is caused by the attached bilateral muscles (m2 of Snodgrass [19]) which originate laterally at the sternite of the abdominal segment 7. The tilting of the gc narrows and widens the lateral hemolymph spaces. In intact animals these movements are clearly visible through the transparent parts of the abdominal sternite 7 and were the first and most obvious indication for the presence of the rhythmic pumping apparatus at the base of the ovipositor valves in *Acheta domesticus*[24].

The gc muscles (m2) are always active in synchrony with the ipsilateral opo8/9 contraction muscles as was evident from long-time recording in more than 25 preparations. However, they can halt or remain in tonic contraction when they contribute to other behavior; the opo8/9 muscles however continue their rhythm at the same time.

### Innervation and rhythm of the accessory hearts

**Figure 7:**
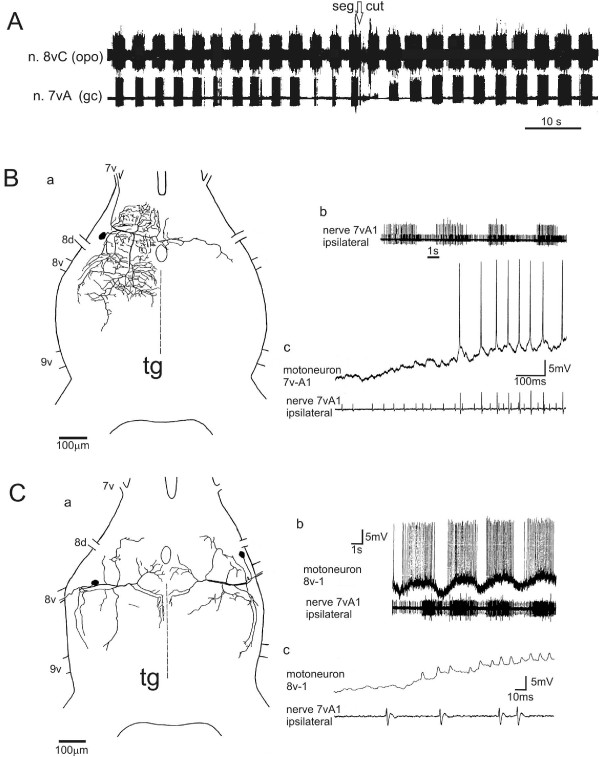
**Morphology and activity of motoneurons that drive the ovipositor pulsatile organs. (A)** Recording from the efferent nerves (suction electrodes) to a opo8 (upper trace, n.8vC) and to the ipsilateral genital chamber muscle (lower trace, n.7vA, gc) before and after disconnection from cns by severing both connectives (seg cut, arrow) posterior to the subesophageal ganglion. The opo8 and gc rhythms persist almost undisturbed. **(B)** a. Branching of one motoneuron for genital chamber movement (7vA-1) in the terminal ganglion (tg, dorsal view) and b. its rhythmic activity along with the second motoneuron to M2 and, c. onset of a burst due to summation of postynaptic potentials elicited by premotor interneurons. **(C)** a. Branching types of two icM8 motoneurons in the terminal ganglion (tg, bilateral, dorsal view) which supply the opo8. b. Rhythmic activity of motoneuron 8v-1. Summation of excitatory postsynaptic potentials originating from premotor interneurons are the basis for bursting activity of the motoneuron. Lower trace shows synchronous activities of the ipsilateral genital chamber motoneurons. c. Expanded view of the onset of a burst.

The terminal ganglion (tg) is a fusion of the abdominal ganglia of the 7^th^ and the more posterior abdominal segments. Its ventral nerves innervate the sternal regions of several segments (7v, 8v, 9v) including the motor innervation of the rhythmic pumping muscles of the opos and the gc. Experiments in which the specific nerve branches that innervate the pumping muscle of an opo are cut selectively show that the contraction is based on neural commands. After denervation it stops immediately and permanently. The pumping muscles of the other opos remain active during these experiments as long as their nerve connection to the tg persists.The rhythmic motor output recorded from the tilting muscles of the gc or the opos typically shows the activity of two excitatory motor units bursting in phase. One unit usually bursts at a higher frequency and longer than the other (Figure 7B, C). The somata of the rhythmic motoneurons for the opos are located ipsilaterally in their respective neuromere. Their neurites exhibit a rich branching, also into the neighboring neuromeres and some even with extensions across the midline (Figure 7B, C).The opo rhythms of intact animals are occasionally accelerated or decreased respectively terminated during certain activities of the abdomen such as oviposition or strong ventilation. Our intracellular recordings from opo motoneurons reveal that the input, which elicits rhythmic motor bursts, starts with distinct excitatory postsynaptic potentials from premotor interneurons (in) which summate and release efferent spikes when the membrane potential rises over the firing threshold of the motoneuron (Figure 7B, C).

### Influences on pattern generation for opos in the terminal ganglion

**Figure 8:**
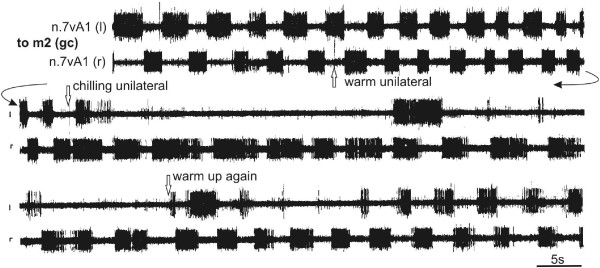
**Influences on the rhythmic bilateral genital chamber movements by unilateral warming and cooling.** Continuous recording of the efferent output to the genital chamber tilting muscles m2 (n.7vA1 l/r). The left-right rhythm accelerates slightly due to unilateral warming of the terminal ganglion (arrow). Cooling the ganglion with a probe on the other side (arrow, chilling unilateral) abolishes the ipsilateral rhythm and slows the antagonist. Recovery to room temperature (22°C, arrow) occurs after 60 s.

**Figure 9:**
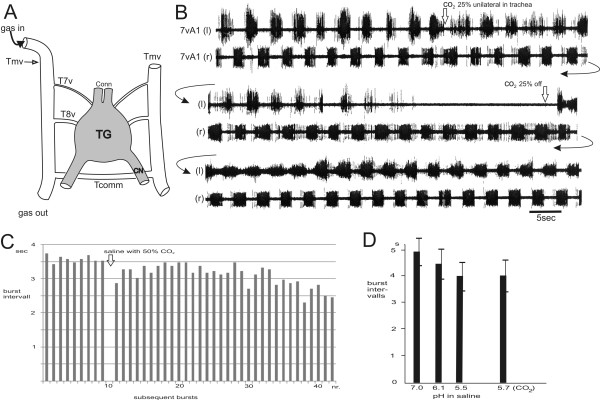
**Altered motor output to the ovipositor pulsatile organs by changing CO**_**2 **_**concentrations in and around the terminal ganglion. (A)** The isolated terminal ganglion (tg) shown with cut connectives (Conn) and cercal nerves (CN) and its supply with tracheal branches (T7v, T8v) originating from the main ventral trachea (Tmv) of the abdomen, one of which is cut and prepared for perfusion with air or an air/CO_2_ mixture (gas in/out). The large commissural trachea (Tcomm) is shown behind the tg. **(B)** Double recording (consecutive traces) of the nerves running to left and right anterior genital chamber muscles (7vA1 l/r) with temporal perfusion of 25% CO_2_ in air through left main tracheal trunk. **(C)** Continuous recording of 7vA1 bursting intervals while applying saline bubbled with a mixture of air and 50% CO_2_.The rhythm accelerates and later deteriorates. **(D)** Average burst durations of rhythmic bursts in nerve 7vA1 during bath application of different pH levels of saline. Frequencies rise (burst duration decrease) with higher acidity due to HCl or 25% CO_2_ diluted in saline. Action potential amplitudes decrease with higher salinity.

The pattern generation for opo pumping originates from the tg. While in intact and freely moving animals the motor output to the pumping muscles can be variable, the auxiliary tilting of the gc by the m2 remains consistently regular after severing the neck connectives and persists for several hours (Figure 7A). The common rhythm of the central pattern generator (cpg) even persists after severance of all peripheral nerves and the anterior connectives of the tg. The cpg thereby becomes separated from all sensory inputs and signals from the other ganglia of the cns. In such an isolated tg, the rhythms remain regular at about 0.5-0.2 Hz in aerated saline. Using this preparation we could also substantiate several non-neural influences on the autonomous rhythm generation and bilateral coordination of the cpg in the tg:(i) Temperature changes applied unilaterally to the tg (Figure 8). By bringing warm or cold Peltier probes close to one side, changes in the left-right alternating rhythm of the opos were provoked. Dramatic inhibitory effects on the rhythm are caused by cooling, which when applied unilaterally decreases and abolishes the ipsilateral motor output of the cpg while the contralateral pattern paces down to a slower rhythm with extended burst durations. Removal of the cold probe gradually restores the initial bilateral rhythm.

(ii) Different concentrations of CO_2_. Infusion of air with gradually increasing pCO_2_ into the lateral trachea that supplies an isolated tg (Figure 9A) slows the ipsilateral rhythmic motor output to the opos progressively and the amplitudes of action potentials decrease (Figure 9B). Finally the rhythm ceases in one hemiganglion while the regular rhythm of the other side persists. Stopping the CO_2_ infusion allows for the rhythm to recover and return to the initial rates. In contrast, when the ganglion surface is superfused with bathing saline in which the pCO_2_ is increased (which also lowers the pH of the saline), the cpg rhythm accelerates and finally transits into more tonic activity (Figure 9C).

(iii) Increasing acidity (with drops of HCl) of the saline bathing of the ganglion. This procedure had an accelerating effect on the cpg for the opo rhythms from 0.23 to 0.26 Hz and also lowered the action potential amplitudes (Figure 9D). A similar effect occurs with an increased pH due to the application of CO_2_ in saline (lowest trace in Figure 9D).

### Interneurons with rhythmic activity for opo muscles

**Figure 10:**
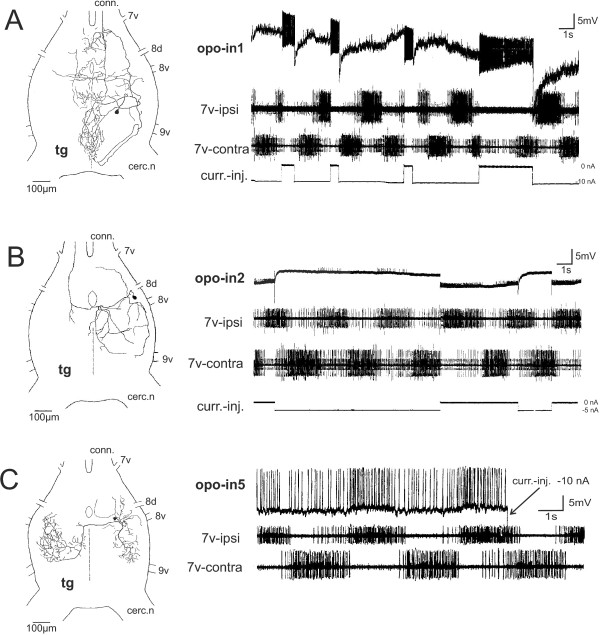
**Morphology of terminal ganglion interneurons and their influence on the ovipositor pulsatile organ and genital chamber rhythm.** Major nerve roots (7v-9v, cerc. n) from neuromeres 7 to 9 are indicated on the terminal ganglion (tg). **(A)** The opo-in1 is a widespread bilateral interneuron with ipsilateral soma and ascending axon collateral showing little rhythm related activity. Intracellular current injection (curr.-inj., lowest trace) elicits an inhibitory effect on the rhythmic output on both sides that is stronger on the ipsilateral motor output to the genital chamber muscles. **(B)** The opo-in2 is a widespread bilateral interneuron extending over three neuromeres (7–9) with opo motor output, a soma in the neuromere 8 and a contralateral ascending axon collateral. Its own rhythmic activity is enhanced when it is depolarized (bridge balance inverted, current injection in lowest trace) and the efferents to the genital chamber muscles are enhanced ipsilaterally and inhibited contralaterally. **(C)** The opo-in5 is a bilaterally branching interneuron with extensive branching in neuromeres 8 and 9. The interneuron bursts in synchrony with the ipsilateral motoneurons but depolarization causes no major changes in rhythm or intensity of motor output.

Five interneurons of the tg that burst in synchrony with the rhythm of the opos (opo-in) were identified by recording and staining. Their morphology differs considerably; further their physiological influence on the opo muscles ranges from transient influences on the rhythm to resetting the rhythm properties. The basic morphological shapes of these interneurons are (Figure 10A-C): (a) local, with branching restricted to the tg and connections between ipsilateral areas of adjacent neuromeres, (b) local, with branches crossing the midline, and (c) intersegmental, with axon collaterals entering anterior connectives and preceding ganglia.

Modifying the activity of the interneurons by electrical stimulation influenced the bilateral motor output to the opos in different ways. Three kinds of affects can be characterized: (i) a transient suppression of the bilateral or only the unilateral motor output (Figure 9A, B). The interneuron opo-in1 has its soma located in the neuromere 9 and branches extensively into all neuromeres but most densely along the median region of the tg. An intersegmental axon collateral ascends in the ipsilateral connective. It exhibits a high tonic spiking activity which can be modulated by irregular bursting. Its effects on the motor output to the opos was most dramatic: when it was released from inhibition the subsequent rebound resulted in intense spiking that inhibits the bilateral motor output to the opos specifically on the side ipsilateral to the soma. Nevertheless, the basic ongoing rhythm for the opos was maintained and not reset by the opo-in1. The interneuron opo-in2 (Figure 10B) has a large soma located in the neuromere 8, and its neurites extend in the ipsilateral neuromeres 8 and 9 and just one branch into the 7^th^ neuromere. The principal axon crosses to the contralateral side, diverges into a smaller posterior branch and then ascends in the contralateral connective to the anterior abdominal ganglion. The opo-in2 bursts in synchrony with the ipsilateral opo motoneurons and when it is hyperpolarized, the ipsilateral opo motoneuron activity is inhibited. That may also slightly affect the basic opo rhythm. (ii) a resetting of the basic rhythm that is achieved by neurons which may be intrinsic to the cpg (published preliminarily as Figure 2E in [18]). The opo-in3, with a dorsal soma located in the 8^th^ neuromere, extends only in the ipsilateral neuromeres 7 and 8. It bursts rhythmically in synchrony with the ipsilateral opo motoneurons. When this rhythm in opo-in3 is abolished by hyperpolarization, the bursting frequency of contralateral opo motoneurons is reduced. Rebounds from inhibition reset the whole opo rhythm starting with ipsilateral excitation and contralateral inhibition. Another interneuron (opo-in4, as APOV-IN4 in [18]) extends ipsilaterally from a particularily posterior and median soma into the 9^th^, 8^th^ and 7^th^ neuromere with some smaller branches crossing over the midline. It bursts in synchrony with the contralateral motoneurons of the opos and has the strongest driving and resetting properties for the opo rhythm. Depolarizations of the opo-in4 cause immediate rhythm reset which inhibits the ipsilateral motoneurons and excites the contralateral motoneurons. (iii) an unaltered rhythm by current injection which is observable in the rhythmically active opo-in5 (Figure 10C). This local interneuron connects bilaterally the 8^th^ and 9^th^ neuromeres with widespread branches. Its activity pattern corresponds with the motor bursts that move the opo and gc muscles ipsilateral to the soma. The motor output is not altered dramatically when this neuron is de- or hyperpolarized.

## Discussion

In the accessory circulatory organs of insects one can distinguish between the pulsatile apparatus and the hemolymph guiding structures which provide for circulation throughout the appendage. In part one of the discussion, we address these two construction elements in the cricket ovipositor circulatory organs with respect to their structure and functional mechanisms and compare them with other accessory pulsatile organs [1-3]. The second part of the discussion is dedicated to the neuroanatomical results and the physiological control of the opos.

### Functional morphology and pumping mechanism

#### The pulsatile part of the opos

**Figure 11:**
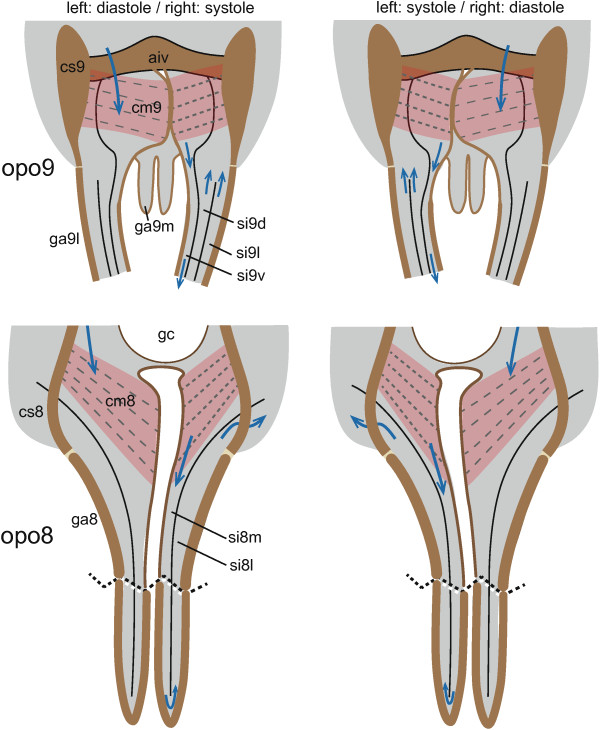
**Functional mechanisms of the ovipositor pulsatile organs.** Organs of abdominal segment 9 (upper graphs) and segment 8 (lower graphs) in two different phases of action. Ipsilateral compressor muscles of both abdominal segments (cm8, cm9) contract simultaneously, alternating with the other side. Thereby they compress the corresponding pumping chamber, i.e. the systole of the organs, and hemolymph is forced in a countercurrent flow through sinuses in the ovipositor gonapophyses (ga) and further back into the abdominal cavity. At the same time the opposite organs are in the diastole phase during which the pumping chambers dilate and fill with hemolymph from the abdominal hemocoel. Simultaneous lateral movements of the genital chamber probably support aspiration of hemolymph during diastole and hinder backflow at systole. Further abbreviations (numbers 8 and 9 refer to the respective abdominal segment, sometimes followed by an additional letter: d, dorsal; l, lateral; m, medial; v, ventral): aiv, anterior intervalvular sclerite; cs, coxosternite; opo, ovipositor pulsatile organ; gc, genital chamber; si, sinus.

The pulsatile part of the opos consists of a pump chamber at the base of each valve (functional scheme Figure 11). The organs of the right and left valve of the same segment have a mirrored but otherwise identical anatomy. The organs of the valves of the 8^th^ and 9^th^ segment function according to the same principle, but have a slightly different anatomy. While the pumps at the base of each ga8 are formed by bulges of flexible cuticle which are compressed by an internal muscle, the pump chambers of the 9^th^ segment are compressed by contractions of an overlying external muscle. Since the compressor muscles of both organs have comparable attachments sites, i.e. laterally at the cs and medially at the flexible cuticle of the base of the ga, they are considered to be serial homologues. Both pumping chambers, as well as the compressor muscles, have not been described in previous anatomical descriptions of the abdominal apex of gryllids (e.g. [19]). The four pumps work continuously whereby the right and left organs of one segment are compressed in alternation. The pumping activities of the 8^th^ and 9^th^ segment are synchronized.

Compared to other accessory pulsatile organs, certain similarities can be found between the functional morphology of the opos and the cercus-hearts in Plecoptera [25]. However, while the cercus-hearts in Plecoptera suck hemolymph out from the cerci into the abdominal cavity, the opos force hemolymph into the valves. Accessory pulsatile organs which likewise force hemolymph into the appendages are the various antenna-hearts; however, they strongly differ in functional morphology and use vessels as hemolymph guiding structures [11,26,27].

#### Circulation within the valves and tracheal ventilation

The systolic compression of the pumping chambers force hemolymph distally into the efferent sinus of the valves. The presence of non-return valves could neither be demonstrated in any ga nor at their bases. Probably backflow is reduced by the narrowing of the proximal bases of the pumping chambers during compression. The hemolymph guiding structures are thin septa of connective tissue which extend the whole length of the ovipositor valves up to their apices. There the septa are perforated enabling the passage of hemolymph into the afferent sinuses. Curiously, only one afferent sinus is present in the ga8, while there are two in the ga9. The diameter of the efferent sinuses is much larger in the opo region than that of the afferent sinuses, which may contribute to slowing any backflow when the pump pressure decreases during diastole.

The hemolymph guiding structures in long abdominal appendages of insects are generally vessels [28]. Longitudinal septa which guide the countercurrent hemolymph flow as in the ovipositor valves have been reported from the thoracic legs, the maxillary and labial palps of many insects [3], and the cerci of the cockroach [4]. While in the legs of many Heteroptera, a rhythmically contracting muscle associated with the septum effectuates a countercurrent circulation within the limb [29,30], in most other insects it is not yet fully understood how the observed countercurrent flows are generated [2,3]. In some appendages without specific muscular pumps, the breathing-related collapse and expansion of tracheae and tracheal sacks cause volume changes that induce hemolymph propagation within the appendage [5,6].

In the cricket ovipositor, the rhythm of the opos is completely independent of ventilatory movements and abdominal compressions. In contrast, *in vivo* observations show that the pulsed hemolymph flow caused by the rhythmic pumping of the opo results in simultaneous collapses of the widened bases of the tracheae within the ovipositor valves. This clearly must enhance the convection of the tracheal gas and thereby the opos also contribute to the O_2_-CO_2_ gas exchange. A similar relationship between circulation and respiration was also found between the wing circulatory organs and the tracheal tubes in the wing veins [31].

#### Simultaneous genital chamber movements

In synchrony with the rhythm of the opos, the apex of the gc moves laterally. We conclude that hemolymph is thereby pressed from the abdomen into the lateral space anterior to the ovipositor base assisting the hemolymph flow toward the ipsilateral ga. Furthermore, the lateral gc movements are probably necessary for hemolymph supply of the entire genitalic region and the abdominal apex since the dorsal heart tube permanently sucks hemolymph away from this region. The gc muscles (m2) always contract in synchrony with the ipsilateral opo8/9. If they contribute to other behavior, e.g. egg laying [32], they can halt or remain in tonic contraction for short periods; the opo8/9 muscles however continue their rhythm in these cases.

#### Neuroanatomy and physiological control

In the fused tg both motoneurons and interneurons of the opos tend to extend over several neuromeres. This morphological feature may functionally ease the intersegmental communication between sensory and motor activity of the adjacent segments, specifically between the rhythmic neurons influencing the pump muscles of the different opos that originate in different neuromeres. Generally, it is rare in insects that the motoneurons innervating non-tergal muscles, such as in the opos, extend with their branches into two or more neighboring ganglia or neuromeres [33]. Basically, interneurons could achieve motoneuron coordination alone when they branch into several neuromeres.

#### Influences on the coordination of the opo rhythm

All contractions of opo muscles are coordinated by neuronal control from a common cpg in the tg. The extent of this neuronal network remains unknown but operates continuously and stably when the ganglion is not addressed by descending neuronal commands.

Higher-order descending interneurons are known to originate in the cricket cns in the subesophageal ganglion serving for the control of respiration [34] and oviposition [35]. Influences on the opo rhythms are evident during strong ventilation or the oviposition procedure when an egg enters the gc and the bilateral muscle pair m2 contracts synchronously [32]. Comparable systems with autonomous and spontaneous neuronal rhythms are known from other isolated insect ganglia which coordinate, e.g. locust respiration [18,36], cricket oviposition [35], and feeding patterns of *Drosophila* larvae [37]. The autonomous cpg rhythms of these systems appear more “natural” than those which require pharmacological or permanent sensory stimulation such as insect walking [38,39], flying [40], and feeding [41,42].

The autonomous and spontaneous cpg for the opos in the cricket tg can be modulated by the following non-neural factors: (i) temperature changes that induce activity changes of the cpg and (ii) lowered pH in the bathing fluid provided by an increased pCO_2_ causing rhythm acceleration. In contrast, when higher levels of pCO_2_ are introduced into the tg via its tracheal supply, the effect is not rhythm acceleration but rather that of an anesthetic. These contrasting CO_2_-effects may reach the cpg in the neuropil by different mechanisms. The rapid effect of pH changes in the bathing fluid may be transferred inward by the glial cells which are interconnected with numerous gap junctions [43]. They may transmit the effect to the cpg neurons for the opos. As an alternative explanation, specific sensory neurons with endings near the surface of a ganglion may monitor pH changes and influence the neurons inside the ganglion – but sensors of this type are so far not known from any insect cns.

The contrasting (non-pH-like) effect of CO_2_ after intra-tracheal infusion inhibits the rhythmic motor output of only the ipsilateral hemiganglion of the tg. This speaks against a pH-effect via the ganglion surface and agrees with the notion that there is no tracheal junction over the midline to the contralateral side of the tg [44]. Apparently the gaseous intratracheal CO_2_ has a low effect on the pH levels in the environment of the cpg neurons of the tg. That seems to indicate a neuronal tolerance to self-produced metabolic CO_2_ in the cns, as was found for single neurons of crickets [45].

The hemolymph that returns from the ovipositor partly overflows the tg with metabolically loaded and more acidic hemolymph caused by a high metabolic rate of the many cuticular sensilla located on the surface of the ovipositor [46]. That may contribute to the regulation of the cpg rhythm as indicated by experimental superfusion of the isolated tg. In this way metabolic requirements may indirectly control the velocity of the hemolymph flow through the ovipositor valves.

#### Coupling of the left-right opo rhythm

Unilateral changes of external influences on the tg, such as cooling, and unilateral application of CO_2_, affect the rhythmic output mainly on the ipsilateral side, at least, for the first minutes of application (Figure 9); the rhythm on the other side remains nearly unchanged. This strong ipsilateral suppression of the motor, and possibly also of premotor neurons, raises the question whether the total ipsilateral cpg is affected. That leads one to assume that the cpg for the opos consists of two (left and right) half centers producing their own – but normally coupled – rhythms.

#### Interneurons and the cpg

All interneurons exhibiting the rhythm of the opos could belong to the cpg itself or are influenced by it. They extend over, at least, two or more neuromeres of the tg. A similarly extensive wiring is required to connect the cpg to the different motoneurons of the segmental neuromeres 7–9 which has efferents to the opos and gc. Yet the exact location of the rhythm-generating neuronal network and the extent of the essential network remain unclear. The “core” of the cpg may be located in the neuromere 8 where all the motoneurons for the rhythmic muscles have branches. At the level of interneurons, only one potentially rhythm resetting interneuron (opo-in3) was found that branches unilaterally in neuromeres 8 and 9, whereas the opo-in4 reaches all neuromeres mainly on one side and the contralateral neuromere 8 [18]. In contrast, the opo-in5 exhibits a morphology that appears well suited for a left-right coordination of all opo-rhythms. However, the physiology of this interneuron, with its ideal left-right connection and rich bilateral arborizations in the neuromeres 8 and 9, is not sufficiently elaborated to substantiate the proposed function.

## Conclusions

Most arthropods have a complex vascular system in which the limbs are supplied with hemolymph by arteries. In insects, this system is greatly reduced and a ventral longitudinal vessel from which such arteries could emanate is lacking [47]. Their thoracic limbs are supplied by sinuses delimited by thin septa of connective tissue which are perforated in the tip region of the appendage enabling a countercurrent hemolymph flow [3]. A comparable condition can also be found in the gonapophyseal appendages in *Acheta*. However, while in most thoracic limbs and cerci it is unclear how the hemolymph flow is generated, a pumping apparatus exists for each of the ovipositor valves. These organs represent evolutionary novelties having a functional morphology which has not been reported from any other auxiliary heart in insects [2]. The origin of the associated pumping muscle must remain unclear since no unambiguous homologization with any of the serial homologues of the abdominal musculature is possible.

With respect to physiological control, it must be emphasized that the neurogenic automatism of the opo is unique among insects. All other known circulatory organs are based on a myogenic automatism which may be neuronally or hormonally modulated [6-10]. The great autonomy of opo rhythm generation is surprising. The only noticeable influence on the cpg interneurons is – apart from general temperature effects and inhibitory cns commands – the pH of the fluid surrounding the tg. This may be linked with the metabolic requirements of the numerous sensilla which are located especially at the ovipositor apex. An additional task of the opos may be the convection of the extensive tracheal system within the ovipositor valves.

From an evolutionary point of view it will be a rewarding task to investigate if corresponding pump organs are associated with the ovipositors in other insects. Future research in this direction could reveal remarkable insights to the evolution of the female ovipositor in insects, a classical topic of comparative morphology in these animals [19,21,22,48-50].

## Material and methods

### Animals

Females of *Acheta domesticus* used in this study originated from breeding stocks in our laboratories. For immobilization the specimens were cooled to 0-4°C previous to and during preparations. All experiments were carried out respecting the relevant ethical guidelines for experimentation with live animals.

### Observation of the pumping organs *in vivo*

The speed and direction of hemolymph flow inside the ovipositor valves is readily recognizable through the transparent regions of the ovipositor cuticle via movement of the hemocytes. Experiments with introducing various vital stains into the hemolymph failed due to immediate clotting that slowed or stopped fluid propagation in the small sinuses of the ovipositor. Observations were made with incident or translucent light under a stereomicroscope. In addition, the pumping action of the opos was video-recorded (camera: Kappa C15) in intact animals (in a small glass chamber from below), as well as from prepared specimens (ventral side up). The range of peak velocities during pumping strokes was calculated (n = 8 preparations) from tracking individual large hemocytes frame by frame in high-speed video sequences (300 fps, Casio Exilim F1) recorded through a dissection microscope in translucent light.

Correlation of the hemolymph pulses in the ovipositor valves to the pumping activity of the opo8 was studied from the ventral side after removal of the subgenital plate that covers the ovipositor base ventrally. The pumping movements of the opo9 system were observed dorsally in semi-intact preparations after removal of overlying muscles and other tissue.

Experimentally induced influences on the opo/gc rhythm were measured in 5–8 animals per parameter, relating the undisturbed burst frequency of the individual preparation with the altered frequency after introducing an influence to the same preparation.

### Morphological methods

Chemical fixation: freshly cut last abdominal segments of female crickets were fixed in alcoholic Bouin (“Dubosq-Brasil” mixture) and subsequently washed in ethanol.

Histological sections: the fixed specimens were embedded after dehydration with acetone in low viscosity resin (Agar Scientific). Serial semithin sections (1 μm thickness) were cut with a diamond knife on an ultramicrotome and stained with a mixture of 1% azure II and 1% methylene blue in a 1% aqueous borax solution for approximately 40 s at 80°C.

MicroCT: a female abdomen fixed in alcoholic Bouin was stained in a solution of 1% iodine in 96% ethanol overnight. After this treatment it was imaged with an Xradia MicroXCT x-ray microtomography system (University of Vienna, Department of Theoretical Biology) with a tungsten source at 60 kVp and 66 μA.

3D reconstruction and visualization: the software Amira 5.4.2 was used for 3D reconstruction of the microCT dataset. Blender (http://www.blender.org) was used to postprocess the meshes exported from Amira and to remodel certain parts using the Amira data as a guide. Images of semi-thin sections were postprocessed with Fiji (http://www.fiji.sc) using the CLAHE plugin to enhance contrast.

### Recording from nerves and muscles

To make preparations of the dorsal side, the median part of the tergites, the gut and the ovaries were removed carefully. That gave access to the tg, peripheral nerves, several muscles of the opos and the gc. The easiest access for recording is to the opo muscles (m2) and its motor nerve 7vA whose bursting activities are always in synchrony with the ipsilateral opo muscles cm8/9. The internal organs were flushed regularly with saline [51]. Care was taken not to block the abdominal spiracles by saline from outside. Extracellular recording was performed with suction electrodes on cut nerve stumps, laterally on intact nerves, or by gently sucking the surface of active muscles near their attachments where movement amplitudes of the fibers were low. The time intervals from the start of a burst to the next burst (myogram or nerve recording) were measured continuously for several hours in more than 25 specimens. In none of these or any of the other 250–300 experiments we found rhythms below 0.2 Hz or above 0.5 Hz at room temperature.

Intracellular recording required a supporting silver platform for the tg. The electrodes for intracellular recording were made of borosilicate glass with 50–80 MΩ tip resistances and had their shaft filled with 1 M LiCl while their tip contained about 1-2% Lucifer yellow in LiCl for iontophoretic staining. Intracellular recording focused on rhythmically active or rhythm-influencing interneurons and motoneurons; the data were stored on magnetic tape (Racal Store 7) or on a PC after digitalization (Datapac K2).

Temperature application (n = 6 preparations): Short metal studs connected to a regulated Peltier element (Peltron, Nürnberg) were brought close to the tg laterally with temperatures of either 0° or 25° Celsius.

Superfusion and infusion of gas mixtures: The different gas mixtures were mixed before application in a gas syringe and each type of experiment was repeated 5 to 8 times.

### Terminology

A confusing multitude of synonyms exist for the ovipositor valves and linked structures (see Scudder [21]). For reasons of comprehensibility we use “gonapophysis” as a descriptive term to refer to all three valves forming the ovipositor shaft in *Acheta* without implying homology. The numbering of some muscles was taken from the descriptions for *Gryllus assimilis* by Snodgrass [19]. The nerve roots of the terminal ganglion were named according to the abdominal segment that they supply, e.g. 8d supplying the dorsal region of the 8^th^ segment and 8v for the ventral region.

## Abbreviations

: Appended numbers 8 and 9 refer to the concerned abdominal segment; cm: Compressor muscle; cns: Central nervous system; cpg: Central pattern generator; cs: Coxosternite; ga: Gonapophysis; gc: Genital chamber; opo: Ovipositor pulsatile organ; opo-in: Ovipositor pulsatile organ interneuron; tg: Terminal ganglion.

## Competing interests

The authors declare that they have no competing interests.

## Authors’ contributions

RH and GP discovered the opos in *Acheta* independently and later combined their efforts. The morphological investigations were carried out by AB, RH, and GP, the neuroanatomical and physiological analysis by RH and MF. The text was written by RH, GP and AB. All authors read and approved the final version of the manuscript.

## Supplementary Material

Additional file 1 **Video.** The clip is divided into three sections. (1) Bases of the gonapophyses 8 are shown in ventral view displaying the left-right alternating pumping of the ovipositor pulsatile organs 8 (subgenital plate removed). The compression of one bulge supports the dilation of the other bulge as it fills up with hemolymph drawn from the abdominal body cavity. Note the alternating lateral shift of the flat cuticular area between the two bulges which narrows the hemolymph connection to the abdominal hemocoel and which probably prevents backflow. (2a) Countercurrent flow of hemocytes in the efferent ga8m (flow direction: left) and the afferent ga8l (flow direction: right), ventral view. The flow pulses alternate between the right (upper one in the video) and left gonapophysis 8 (2b) Collapsing tracheae in the proximal gonapophysis 8, ventral view. (3) Dorsal view of the muscles of the ovipositor pulsatile organ 9, which are active in the alternating pumping movements visible below the terminal ganglion (overexposed; cercal nerves cut). The whitish processes at the end of ovipositor pulsatile organ 9 are the median gonapophyses 9. Click here for file
